# Environmentally Friendly Hydrothermal Processing of Melon by-Products for the Recovery of Bioactive Pectic-Oligosaccharides

**DOI:** 10.3390/foods9111702

**Published:** 2020-11-20

**Authors:** Xiana Rico, Beatriz Gullón, Remedios Yáñez

**Affiliations:** Department of Chemical Engineering, Faculty of Science, University of Vigo (Campus Ourense), As Lagoas, 32004 Ourense, Spain; xianarico@protonmail.com (X.R.); bgullon@uvigo.es (B.G.)

**Keywords:** melon by-products, hydrothermal treatment, pectin-derived oligosaccharides, oligogalacturonides, phenolic compounds, antioxidant activities

## Abstract

Melon by-products, that currently lack high value-added applications, could be a sustainable source of bioactive compounds such as polysaccharides and antioxidants. In this work, melon peels were extracted with water to remove free sugars, and the water-insoluble solids (WISs) were subjected to hydrothermal processing. The effect of temperature on the composition of the obtained liquors and their total phenolic content was evaluated. The selected liquors were also characterized by matrix assisted laser desorption/ionization-time of flight mass spectroscopy (MALDI-TOF MS), fourier transform infrared spectroscopy (FTIR) and high performance anion exchange chromatography with pulsed amperometric detection (HPAEC–PAD), and its phenolic compounds were identified and quantified by high-performance liquid chromatography-diode array detector-tandem mass spectrometry (HPLC–DAD–MS/MS). In addition, the spent solids from the hydrothermal treatment were characterized and their potential use was assessed. At the optimal conditions of 140 °C (severity 2.03), the total oligosaccharide yield accounted for 15.24 g/100 g WIS, of which 10.07 g/100 g WIS were oligogalacturonides. The structural characterization confirmed the presence of partially methyl esterified oligogalacturonides with a wide range of polymerization degrees. After precipitation, 16.59 g/100 g WIS of pectin were recovered, with a galacturonic acid content of 55.41% and high linearity.

## 1. Introduction

Around 1300 million tonnes of food are discarded every year, between production and consumption [1]. For instance, in Europe alone, 88 million tonnes of food waste were generated in 2012, of which about 39% arises from manufacturing activities [2,3]. It would be equivalent to 20% (*w*/*w*) of the total food produced and a generation of 173 kg of food waste per person per year [3].

In particular, waste and by-products from the fruit and vegetable processing industries, which are characterized by a high fraction of discards, are in fifth place in the ranking of total food waste in Europe, assuming 8% of the total [4,5]. These wastes, with high contents of moisture and microbial loads, have an important contribution to the global environmental burden, and currently, they are mainly destined for low value-added applications, such as composting or animal feed [4,5]. In addition, the requirements of European regulations regarding the prevention, management and landfill of waste (Directives (EU) 2018/851 and (EU) 2018/850), support the growing need to find alternatives for their valorization that allow them to be re-incorporated into productive processes as raw materials, thus adopting the principles of circular economy and the concept of industrial symbiosis [6,7].

In this context, one of the most consumed fruit crops worldwide is melon (*Cucumis melo* L.) [8]. According to information provided by the Food and Agriculture Organization of the United Nations (FAO), its annual production (*Cucumis melo* L.) has recorded slight fluctuations over the last ten years, between 25.5 and 27.3 million tonnes [9]. It is often used for minimal fresh processing as cylinders, cubes or slices [10] or for the production of juices, jams, compotes or dehydrated pulp [11,12]. The processing by-products account for around one third of the weight of the fruit and would be mostly made of skin and lower percentages of pulp and seeds [13].

Melon by-products have high contents of polysaccharides and minerals, as well as bioactive compounds with antioxidant activity, including phenolic compounds such as flavonoids and phenolic acids, with important applications in several food and pharmaceutical industries [14,15,16,17,18]. One of the main components of the melon peel is pectin [19,20], a complex heteropolysaccharide found in the plant cell walls of many agro-industrial by-products, consisting of a backbone of galacturonic acid (GalA) with alternating ‘smooth’ and ‘hairy’ regions [21,22]. Homogalacturonans (HGs) represent the smooth regions, which are composed of GalA linked by α-(1,4) glycosidic bonds and constitute about 65% of the pectin, while rhamnogalacturonans (RG-I and RG-II) form the hairy region, with RG-I being the main branched structure of pectin, constituting 20–35% of the molecule [21]. In RG-I, the GalA backbone is often interrupted by rhamnose units bearing neutral sugar side chains composed mainly of galactose and arabinose [22]. This polymer displays important applications in the food industry as thickener, gelling agent, stabilizer, encapsulant and edible food packaging films [23,24,25]. Moreover, pectin-derived oligosaccharides are reported as emerging prebiotics and in recent years they are being studied for their potential health benefits [21,23,26].

In the last decade, autohydrolysis has been applied successfully for the extraction of pectic oligosaccharides from several fruit by-products, such as lemon peels [27], orange peels [28], sugar beet pulp [29], pomegranate peels [30] or mango peels [31]. The hydrothermal processing, carried out in pressurized hot water, is catalyzed by the hydronium ions of the reaction media as well as by the acids generated in situ. This green technology allows the selective solubilization of hemicelluloses, yielding a pre-treated solid fraction enriched in cellulose and lignin, suitable for further applications in a bio-refinery context [32,33,34,35]. Temperature and time are the main factors affecting this treatment, and their effect can be measured by combining the two variables into a single parameter called the severity factor (*R*_0_) [33].

The application of bioactive compounds in the formulation of novel functional foods has been drawing the attention of researchers due to the health benefits associated with their consumption and the high demand and acceptance that they nowadays are having in the market [36,37,38]. Melon by-products, that currently lack high value-added applications, could be considered as a cheap and sustainable source of these compounds [5,39]. However, the literature concerning extraction technologies for the production of bioactive compounds from melon by-products is limited. For instance, the extraction of melon peel pectin with citric acid has been optimized recently by Muthukumaran et al. [20]. In another study, Raji et al. [19] assessed the influence of several acids on the extraction of melon peel pectin, obtaining the highest pectin yield (29%) with citric acid. Several studies focused on the recovery of the phenolic fraction by conventional alcoholic extraction [8,14,40], aqueous wet grinding [41] or ultrasound assisted extraction [42] have also been reported. More recent studies [11,43] evaluated the extraction of bioactive compounds (total phenolics, chlorophylls, total carotenoids and vitamin C) from several melon parts by ultra-turrax homogenization in alcoholic media.

However, to the best of our knowledge, despite the important content of pectin of the melon peels, no literature dealing with the extraction and characterization of pectin-derived oligosaccharides with phenolic content from these by-products is available. Hence, the need for additional research focused on its extraction and characterization, since the polysaccharide structure has an important role in their functionality and applications [23,44].

Therefore, this work evaluates the suitability of non-isothermal autohydrolysis for the production of melon by-products extracts enriched in functional pectic oligosaccharides with antioxidant activity. The liquid phases obtained were assayed for their chemical composition, total phenolic contents and antioxidant activities (by DPPH (α,α-diphenyl-β-picrylhydrazyl), ABTS (2,2-azino-bis-3-ethylbenzothiazoline-6-sulphonic acid) and FRAP (ferric reducing antioxidant power) methods). The identification and quantification of the main phenolic compounds was also performed by HPLC–DAD–MS/MS. Additional information about the pectic oligosaccharides composition has also been provided by FTIR and MALDI-TOF. Finally, in order to propose further bio-refinery stages, the spent solids were characterized and their susceptibility to enzymatic hydrolysis was also studied.

## 2. Materials and Methods

### 2.1. Standards

The standards used for determination were: arabinose (99%), xylose (99%), rhamnose monohydrate (99%), galacturonic acid monohydrate (97%), acetic acid (96%), formic acid (99%), gallic acid (97.5%), trolox (97%), digalacturonic acid (85%), trigalacturonic acid (90%) and polygalacturonic acid (90%), purchased from Sigma-Aldrich (Steinheim, Germany); glucose (98%), purchased from Scharlau (Barcelona, Spain); galactose (99%), mannose (99%) and fructose (98%), purchased from Panreac (Barcelona, Spain). For the identification of phenolic compounds, 4-hydroxybenzoic acid (99%), p-coumaric acid (98%), gallic acid (97.5%), salicylic acid (99%), tyrosol (98%), sinapic acid (98%) and caffeic acid (98%) were purchased from Sigma-Aldrich (Steinheim, Germany); ferulic acid (99%) and vanillic acid (97%) were purchased from Sigma-Aldrich-Fluka (Steinheim, Germany).

### 2.2. Raw Material

Melon by-products (var. piel de sapo) consisting of peels with a small amount of seeds were kindly supplied by FreshCut, S.L. (Vigo, Pontevedra, Spain), a company dedicated to the development and marketing of fresh cut products. They were cut into small pieces and frozen at −18 °C until use.

### 2.3. Aqueous Extraction of Melon by-Products

Melon by-products (MPs) were centrifuged (SV4028, AEG Electrolux) at 2800 rpm for 15 min, separating the extracts from the solid fraction. The solid fraction was then washed with distilled water for 15 min at room temperature in a 2 L stirred reactor (20 g of water/g of oven dry MP), and centrifuged again for 15 min. The resulting water insoluble solids (WISs) were ground in a regular coffee grinder and then frozen until use, except a small fraction that was dried in an oven at 60 °C for analysis.

### 2.4. Autohydrolysis

**Figure 1:**
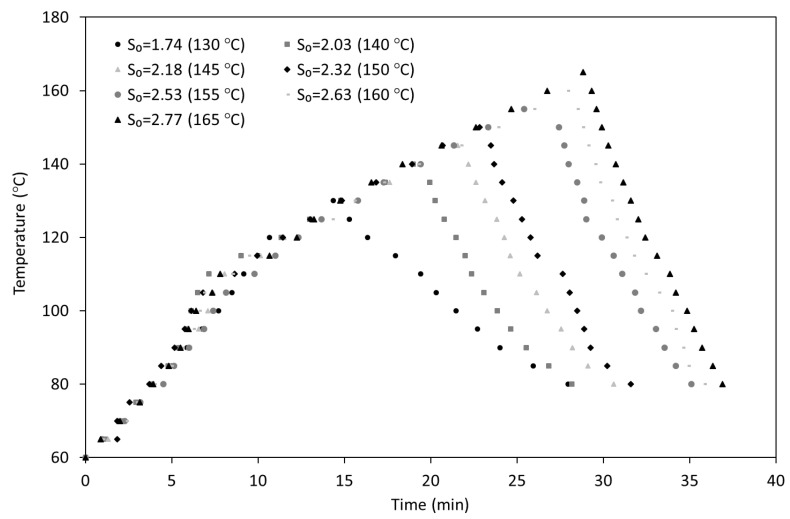
Heating and cooling temperature profiles determined for the autohydrolysis experiments carried out at all conditions assayed.

Wet WIS samples were mixed with water in a liquid to solid ratio of 20 kg/kg (oven dry basis) in a 0.6 L stainless steel reactor (model 4842 from Parr Instruments, Moline, IL, USA). Based on the literature concerning pectin-rich raw materials [27,28,29] and on preliminary experiments (data not shown), the hydrothermal treatments were performed under non-isothermal conditions in the range of temperatures from 130 to 165 °C. Figure 1 displays the temperature profiles followed during the heating and cooling of all the experiments carried out in this work. With the purpose of facilitating the comparison between different reactors, the combined effects of temperature and time can be expressed in terms of the severity (*S*_0_). This parameter is defined as the logarithm of the severity factor *R*_0_ [45] and it is calculated by the following equation:

(1) $$\begin{array}{cc} S_{0}=\log R_{0} & =\log\left(R_{0}{}_{HEATING}+R_{0}{}_{COOLING}\right)\\ & =\log\left(\int_{0}^{t_{Max}\;}\exp(\frac{T\left(t\right)-T_{REF}}{\omega })\times \mathrm{dt}+\int_{t_{Max}}^{t_{F}}\exp\left(\frac{\;T\prime \left(t\right)-T_{REF}}{\omega }\right)\times \mathrm{dt}\right) \end{array}$$

where *t_Max_* is the time (min) needed to achieve the target temperature of each treatment (*t_Max_*, °C); *t_F_* is the time (min) of cooling; T(t) and T’(t) (°C) represent the temperature profiles in the heating and cooling stages, respectively; and ω and *T_REF_* are parameters whose values are fixed according to the literature (ω = 14.75 °C; *T_REF_* = 100 °C). The calculated values of *S*_0_ for the performed experiments are also included in Figure 1.

After each autohydrolysis treatment, the solid and liquid phases were separated by manual pressing using a cloth bag. The liquid phases were weighed to determine liquor recovery and stored at 4 °C until further analysis, and the solid phases were washed with water, dried in an oven at 60 °C, weighed and subjected to moisture determination in order to calculate the solid yield. Aliquots of the autohydrolysis liquors and the spent solids of the optimal treatment were analyzed using the methodology described below.

### 2.5. Pectin Precipitation

Ethanol was added to the autohydrolysis liquor obtained at the optimum conditions to a final concentration of 75% *v*/*v*. After mixing, the solution was allowed to stand at 4 °C overnight, after which it was centrifuged (Rotixa 50 RS centrifuge, Hettich Lab Technology, Tuttlingen, Germany) at 4000 rpm (radius of 211 mm) for 20 min and subsequently washed with 96% ethanol. After separation by centrifugation, the washed precipitate was oven dried at 55 °C until constant weight and redissolved in water for analysis.

### 2.6. Enzymatic Hydrolysis of Autohydrolyzed Solids

The dried spent solids from hydrothermal treatment at the selected conditions were treated by enzymatic hydrolysis to assess their digestibility. The enzyme cocktail used contained endopolygalacturonase (Viscozyme L from *Aspergillus aculeatus*, 50 U/g spent solid), cellulase (Celluclast 1.5 L, 12 filter paper unit (FPU)/g spent solid) and β-glucosidase (Novozym 188, 5 international unit (IU)/FPU cellulase). The enzymes used in this study were kindly supplied by Novozymes, Madrid, Spain. The hydrolysis was carried out at 37 °C and pH = 5 for 48 h.

### 2.7. Analytical Methods

#### 2.7.1. Analysis of the WISs and Spent Solids from Autohydrolysis Treatment

The dried WISs and solids from hydrothermal treatment were subjected to moisture (TAPPI T-264-om-88 method) and ash (T-244-om-93 method) determination. Protein was calculated using the nitrogen content (6.25 g protein/g nitrogen), which was determined with a Thermo Fisher Quest Flash EA 1112 analyzer, using 130 and 100 mL/min He and O_2_ and an oven temperature of 50 °C. Metals were analyzed using a fast sequential atomic absorption spectrometer after digestion in an MLS-1200 Microwave Labstation mega with 5 mL of HNO_3_ 65% (*w*/*w*), 1 mL of H_2_O_2_ 30% (*w*/*v*) and 0.5 mL of HF 40% (*w*/*w*). Conventional quantitative acid hydrolysis with 72% (*w*/*w*) H_2_SO_4_ (TAPPI T13 m method) was used to determine the content of hemicelluloses, glucan and lignin. The solid residue from hydrolysis was recovered by filtration, oven-dried and considered as Klason lignin (TAPPI T13 m assay), and the liquid phase was analyzed for monosaccharides (rhamnose and arabinose) and acetic acid (coming from acetyl groups) by high-performance liquid chromatography (HPLC) using an Agilent 1260 equipped with a refractive index (RI) detector with an Aminex HPX-87H column (BioRad, Life Science Group, Hercules, CA, USA) operating as follows: mobile phase, 3 mM H_2_SO_4_; flow, 0.6 mL/min; temperature, 50 °C. Another separation with an Aminex HPX-87P column (BioRad, Life Science Group, Hercules, CA, USA) was performed for the determination of glucose, xylose, galactose, arabinose and mannose, operating with deionized water as the mobile phase, at a flow rate of 0.4 mL/min at 80 °C. The arabinose content was calculated as the average of the results obtained by both columns. The liquid phase was also subjected to uronic acid determination by spectrophotometry using galacturonic acid as a standard for quantification [46].

#### 2.7.2. Chemical Characterization of Liquors

The samples of liquors from hydrothermal treatments were filtered through 0.45 μm membranes and used for the direct HPLC determination of galacturonic acid, rhamnose, arabinose, formic acid and acetic acid using the same method employed in the analysis of the WIS fraction with an Aminex HPX-87H column. Another aliquot of filtered liquors was quantified for glucose, xylose, galactose, arabinose, mannose and fructose using an Aminex HPX-87P as described for the WIS fraction. For the determination of OGalA (oligogalacturonides), another sample of liquors was subjected to total enzymatic posthydrolysis using cellulases (Celluclast 1.5 L) and an endopolygalacturonase (Viscozyme L from *Aspergillus aculeatus*) at 37 °C and pH = 5 for 40 h, with a cellulase loading of 5 FPU/g liquor and an endopolygalacturonase loading of 45 U/g liquor [27,47]. The neutral oligosaccharides were quantified by quantitative posthydrolysis (4% (*w*/*w*) sulphuric acid at 121 °C for 20 min). The reaction products of both posthydrolyses were assayed by the same HPLC methods using the already mentioned columns. The increase in the concentrations of monosaccharides and acetic acid caused by posthydrolysis provided a measure of the oligomer concentration and their degree of substitution by acetyl groups (Ac-O). OSs (oligosaccharides) were expressed as monosaccharide equivalents. The content of non-volatile compounds (NVCs) was measured by oven-drying at 60 °C until constant weight. Other non-volatile compounds (ONVCs) were calculated by difference as (NVCs−monosaccharides−oligosaccharides)/NVC. Protein and ash were determined using the same methods as for the solid fractions. All determinations were made in triplicate.

#### 2.7.3. Total Phenolic Content (TPC)

The total phenolic content of autohydrolysis liquors was determined according to the Folin–Ciocalteau method [48]. Aliquots of the samples (500 µL), water as blank and gallic acid standards (10–80 mg/L) were mixed with water (3.75 mL), and Folin–Ciocalteu chemical (1:2 *v*/*v*; 250 µL) and Na_2_CO_3_ (10% *w*/*v*; 500 µL). These mixtures were kept in darkness at room temperature for 1 h and their absorbance was then measured at 765 nm. The results were expressed as mg of gallic acid equivalents (GAE)/g dried WISs. All samples were analyzed in triplicate.

#### 2.7.4. Antioxidant Activity

Antioxidant capacity was evaluated using three different methods, namely DPPH (α,α-diphenyl-β-picrylhydrazyl), ABTS (2,2-azino-bis-3-ethylbenzothiazoline-6-sulphonic acid) and FRAP (ferric reducing antioxidant power) according to the methodology described by Gullón et al. [49]. In all antioxidant capacity assays, Trolox was used as a standard and results were expressed as mg Trolox equivalents (TE)/g dried WISs as the mean of three replicates.

#### 2.7.5. Identification and Quantification of Major Phenolic Compounds Using HPLC–DAD–MS/MS

The profile of phenolic compounds was analyzed using an Agilent model 1260 Infinity (Palo Alto, CA, USA) connected directly to a mass detector AB SCIEX Triple Quad 3500 (AB Sciex, Foster City, CA, USA) equipped with a turbo V™ electrospray ionization source (ESI), and to a Waters 996 photodiode array detector. The separation was carried out on a Phenomenex Luna C18 column (150 mm × 2 mm; 3 μm), with an injection volume of 5 μL and a flow of 300 μL/min. The mobile phase was 0.1% (*v*/*v*) formic acid in water (A) and 0.1% (*v*/*v*) formic acid in acetonitrile (B). The gradient used was: 98% A (*v*/*v*), 0–4 min; 98–80% A (*v*/*v*), 4–7 min; 80–10% A (*v/v*), 7–14 min; 10% A (*v/v*), 14–15 min; 10–98% A (*v/v*), 15–17 min. The mass spectrometer was run in positive and negative ionization, using N_2_ as the nebulizer and collision gas, with an ion spray voltage of 4500 V, source temperature of 400 °C and nebulizer gas pressure of 55 psi. The compounds were identified by the comparison with the corresponding standards, based on retention time data, extracted ion chromatograms and MS/MS spectra. For the quantification, calibration curves were made by using different concentrations of the standards and plotting them against peak areas.

#### 2.7.6. Structural Characterization of the Extracted Oligogalacturonides (OGalA)

To obtain detailed information on the structural characteristics of solubilized oligogalacturonides at the optimal temperature of autohydrolysis, the liquors were freeze-dried and were analyzed using different analytical techniques including FTIR, HPAEC-PAD and MALDI-TOF.

##### Fourier Transform Infrared Spectroscopy (FTIR)

The FTIR analysis of the oligogalacturonides was performed on a Nicolet 6700 Spectrometer. A total of 34 scans were accumulated in transmission mode with a resolution of 4 cm^−1^. The spectrum was obtained in a range of 4000–400 cm^−1^.

##### High Performance Anion Exchange Chromatography with Pulsed Amperometric Detection (HPAEC–PAD)

The oligogalacturonides were analyzed by HPAEC–PAD using an ICS3000 chromatographic system (Dionex, Sunnyvale, CA, USA), fitted with a CarboPac PA-1 column (2 mm i.d. × 250 mm) in combination with a CarboPac PA guard column (2 mm i.d. × 25 mm) and an ISC3000 PAD detector. HPAEC–PAD was performed following the pectic oligosaccharides (POS) identification method described by Gómez et al. [27]. GalA with DP 1, 2 and 3 were used as standards, as well as a mixture of OGalA prepared by the hydrolysis of a 1% (*w/w*) solution of commercial polygalacturonic acid at 121 °C for 40 min at pH = 4.4 adjusted with NaOH [27].

##### Matrix Assisted Laser Desorption/Ionization-Time of Flight Mass Spectroscopy (MALDI-TOF MS)

The absolute masses of oligogalacturonides were obtained by MALDI-TOF MS, using an Ultraflex workstation (Bruker Daltonics, Billerica, MA, USA), operating in reflectron mode and positive polarity. Sample preparation was performed according to the protocol described by Gómez et al. [50] using 2,5-dihydroxybenzoic acid (DHB) as the matrix. Data were acquired and processed by means of the Flex Control and Flex Analysis software (Bruker Daltonics, Billerica, MA, USA), respectively.

## 3. Results and Discussion

### 3.1. Chemical Characterization of the Raw Material

As is the case for other food by-products, the MPs are characterized by a high moisture content (90.05%). In order to eliminate free sugars and other water-soluble compounds, which would decompose into undesirable compounds during the hydrothermal treatment, a centrifugation stage and aqueous extraction followed by further centrifugation was proposed. The chemical composition of the MP extracts and water insoluble solid (WIS) fractions obtained is shown in Table 1. The extractives accounted for 42.21% of the raw material and contained mostly sugars (especially glucose and fructose) and some protein and organic acids. The remaining WISs (57.79% of the raw material) were mainly made up of glucan (includes glucose from cellulose and other glucose moieties) and Klason lignin (24.54 and 19.96%, respectively), followed by protein and galacturonan (11.36 and 11.99%), and minor amounts of other polysaccharides or substituents. Ash and some minerals were also determined, highlighting the content of potassium and magnesium. In general, the presented data are in range of that found in recent literature for melon peels [8,15,16,51]. However, the results showed sample variability and reported clear differences in the chemical composition, for instance, in the protein and ash contents (ranges from 3.25 to 19.14% and from 3.67 to 11.13%, respectively). They are thought to be related with the variety of plant used, the state of ripening of the fruit and/or the analytical methodology used in each case.

Compared with other pectin-rich raw materials that have also been used for pectic oligosaccharides extraction, such as lemon and orange peels, MPs contain a similar glucan content, considerably higher proportions of lignin and protein as well as a lower amount of galacturonan [27,28]. However, the sugar beet pulp presented comparable protein content but a much higher percentage of arabinosyl substituents and lower galacturonan and lignin [29].

### 3.2. Effect of Hydrothermal Processing on Autohydrolysis Liquors

WIS samples were subjected to non-isothermal autohydrolysis at maximum temperatures in the range of 130–165 °C or *S*_0_ between 1.74 and 2.77, according to temperature profiles shown in Figure 1. This treatment mainly causes the solubilization of the pectin, protein and other hemicellulosic fractions as well as low molecular weight phenolic compounds, yielding spent solids with increased proportions of glucan and lignin [27,28,29,30]. Therefore, as expected, in this study, the percentage of solubilization of WISs increased with the temperature of the treatment reaching a maximum value of 36.46 g/100 g WIS in the experiment performed at 165 °C. A similar trend has been previously observed during the autohydrolysis treatment of lemon and oranges peels, reaching higher percentages of solubilization in both cases, with values close to 50% and greater than 60%, respectively, under the same operational conditions [27,28].

Depending on the severity of the treatment, the autohydrolysis liquors contain different proportions of high molecular weight polymers, oligomers as well as monomers and sugar degradation products, among others. The degree of polymerization and the substitution pattern of the extracted oligosaccharides are also affected by the treatment severity. It is well known that the structural attributes play an important role, since it is evident that the chemical and physicochemical features can affect, for instance, the cholesterol-lowering ability, prebiotic potential or emulsifying properties [21,23]. Therefore, the successful modulation of the treatment severity can be used to obtain tailored oligosaccharides suitable for industrial/food applications and to limit carbohydrate decomposition or lignin solubilization, which would result in the presence of undesired compounds in liquors.

#### 3.2.1. Liquor Composition

Most of the autohydrolysis soluble products are non-volatile compounds (NVCs), which are quantifiable by oven-drying the liquors. These NVCs are made of oligosaccharides, monosaccharides, organic acids and other compounds (ONVCs). This fraction includes protein, protein-derived compounds, soluble minerals, and acid soluble lignin.

**Figure 2:**
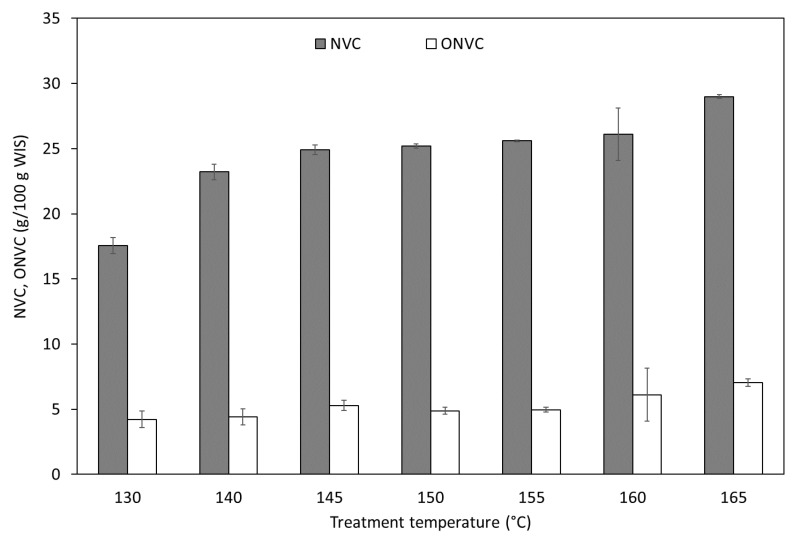
Temperature dependence of the concentration of NVCs and ONVCs in the reaction liquors. NVC, non-volatile compound; ONVC, other non-volatile compound or impurity.

Figure 2 shows the severity dependence of the mass fraction of NVCs and ONVCs in the autohydrolysis liquors (expressed as g per 100 g WIS treated). The lowest NVC content was noted at 130 °C, and then this parameter gently increased in experiments carried out at temperatures between 140 to 165 °C, with values between 23.22 and 28.99%. Regarding ONVCs, in this kind of raw materials when operating at low to moderate severities, this fraction would contain mostly protein and soluble minerals. However, as can be seen in Figure 2 when the autohydrolysis was performed at 165 °C, higher NVC and ONVC contents have been detected in the reaction media. This fact would be related to the increase in the solubilization of WISs and to the presence of decomposition reactions, which would result in the generation of undesirable compounds, such as furans or organic acids, also reported in previous studies [27,52]. Oligosaccharides made the most part of the NVC at all the treatment temperatures, fluctuating between 59 and 66% of this fraction at temperatures of 130–155 °C and then decreasing down to 54% at 165 °C, due to further depolymerization and degradation reactions. In addition, other components present in the NVC of all the autohydrolysis liquors are monosaccharides (in the range of 8–10 g per 100 g NVC) and organic acids (6–11 g per 100 g NVC).

The ratio of total OSs to ONVCs increased from 2.5 to 3.5 at temperatures from 130 to 140 °C and then gradually decreased down to 2.2 at 165 °C, similarly to the results reported for lemon and orange peels and sugar beet pulp, where this ratio decreased from 4 or 5 at the lowest temperatures assayed to 1 or 0 at the most severe ones [27,28,29].

**Figure 3:**
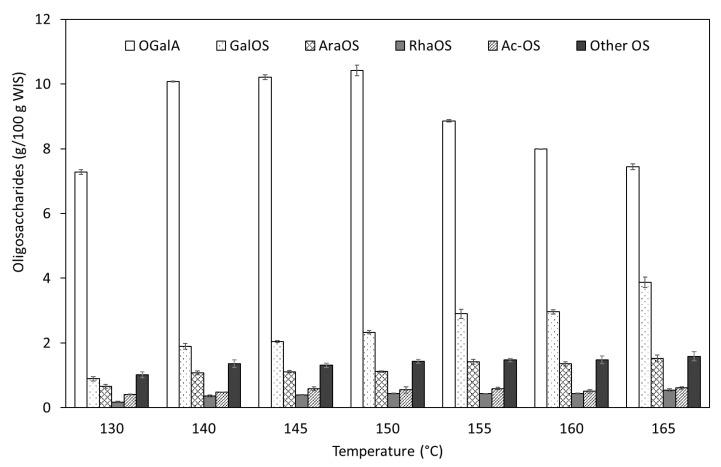
Temperature dependence of the oligosaccharides. OGalA: oligogalacturonides; GalOS: galactose units in oligosaccharides; AraOS: arabinose units in oligosaccharides; RhaOS: rhamnose units in oligosaccharides; Ac-OS: acetic acid units in oligosaccharides; OS: oligosaccharides.

In order to provide additional information about the chemical composition of autohydrolysis liquors, Figure 3 shows quantitative information on oligosaccharides (determined as monosaccharide equivalents and expressed as g per 100 g WIS treated). The total oligosaccharides content increased with the severity of the treatment up to reach a maximum of 16.28 g/100 g WIS in the experiment performed at 150 °C. As can be observed in Figure 3, oligogalacturonides (OGalA), the main component of the liquors, showed a similar pattern, with the highest contents being between 140 and 150 °C. They exhibited a maximum of 10.41 g/100 g WIS at 150 °C, corresponding to a conversion yield of 80.02% (g of OGalA monomer equivalents in liquors/100 g of galacturonan monomer equivalents in the WISs). As expected, higher severities led to liquors with decreased OGalA suggesting their depolymerization. Other oligosaccharides detected were mostly GalOS (galactose units in oligosaccharides), followed by AraOS (arabinose units in oligosaccharides), and smaller contents of glucose, acetic acid, mannose, rhamnose and xylose units in oligosaccharides (GOS, Ac-OS, ManOS, RhaOS and XOS, respectively). The temperature dependence of GalOS, AraOS, RhaOS and Ac-OS is shown in Figure 3, whereas the sum of ManOS, XOS and GOS by simplicity has been included in the other oligosaccharides fraction. In general terms, the percentages of GalOS, AraOS, RhaOS and Ac-OS gradually increased in all the temperature range evaluated, reaching values of 3.87, 1.53, 0.55 and 0.61 g/100 g WIS, respectively, at 165 °C. However, other compounds such as GOS have experienced only slight variations, remaining close to an average value of 0.56 g/100 g WIS in all the conditions assayed.

In this study, galacturonan presented higher susceptibility to hydrolysis reactions than other polymers which required greater treatment severity. For instance, Galacturonan into OGalA, Galactan into GalOS and Arabinosyl S. into AraOS conversions close to 60% were obtained in experiments carried out at 130, 150, and 140 °C, respectively. A maximum temperature of 165 °C was required to reach a complete conversion of Galactan into GalOS, whereas in the case of OGalA the maximum was obtained at 150 °C (80.02%). Similar patterns have been found for lemon and orange peels and sugar beet pulp, with the highest OGalA solubilization at temperatures between 150 and 160 °C, whereas higher severities were needed for the optimum extraction of other oligosaccharides [27,28,29].

The selectivity of the autohydrolysis treatment was confirmed by the low conversion yield of glucan into GOS which oscillated around 2% for all the severities assayed, therefore leaving solid fractions with interesting proportions of glucan and applications in further biorefinery stages. Other studies working with higher maximum temperatures found an increasing GOS yield with increasing temperature [27,28,29]. The total oligosaccharides to monosaccharides ratio, with values between 6 and 9 (with the maximum at 150 °C), also gives insight into the selectivity of the process. The monosaccharides (MS) fraction showed small variations in the interval studied, with an average value close to 2 g/100 g WIS. It was mainly constituted by fructose and glucose, followed by galactose and arabinose (coming from arabinan, galactan or arabinogalactan) and some xylose. There is a lot of variation in the OS/MS ratios found in the literature for liquors rich in pectic oligosaccharides recovered by autohydrolysis. For instance, lemon peel liquors had ratios decreasing from 6 to 2 when increasing the treatment temperature [27], while orange peel and sugar beet pulp liquors had much higher ratios with maximums of 16 and 47, respectively, both at a treatment temperature of 150 °C [28,29]. Formic acid was the main degradation product generated from glucose and fructose degradation, whose production increases with the treatment temperature up to 0.72 g/100 g WIS at 165 °C. This value is considerably higher than those found at the same temperature in sugar beet pulp autohydrolysis liquors (0.38 g/100 g sugar beet pulp) [29], lemon peel waste liquors (about 0.16 g/100 g WIS) [27], and orange peel liquors (about 0.21 g/100 g WIS) [28].

The amount of liquors recovered was also influenced by the treatment severity. At 130 °C only a 75% (*w*/*w*) of the liquors were recovered, lowering the yields obtained. At temperatures from 140 to 160 °C this parameter reached a value of about 85% and lastly at 165 °C it increased to 90%. Previous studies with similar raw materials related higher water retention capacity with the pectin content of the solids [27,28], therefore, increased liquor recovery would be associated with increased pectin fraction solubilization.

Taking into account that there are no important differences in the results obtained for the recovery of OGalA in the temperature range between 140 and 150 °C, the lowest temperature, 140 °C, was chosen as the optimum treatment temperature, resulting in an average OGalA content of 10.07 g/100 g WIS, corresponding to a yield of 77.42% and a total oligosaccharide content of 15.24 g/100 g WIS. In this study, a lower operating temperature allowed higher yields than the ones achieved in the optimum for water insoluble substrates of orange peels (66.23% at 160 °C) and sugar beet pulp (56.77% at 160 °C) and similar to that in water insoluble substrates of lemon peels (79.32% at 160 °C). However, due to a minor presence of galacturonan in the raw material, the resulted OGalA content was lower for MPs than for lemon, orange and passion fruit peels (18.28, 13.96 and 13.78 g/100 g WIS), sugar beet pulp, two different cultivars of pomegranate peels and pomelo peels (12.06, 13.58, 14.71, 15.04 g/100 g raw material, respectively) [27,28,29,30,53,54]. There have also been studies that yielded a lower OGalA content, probably due to a lower pectin content in the raw material: 8.46 and 6.49 g/100 g raw material for apple pomace and cacao pod husks and 7.82 g/100 g WIS for jackfruit peels [55,56,57]. Under the selected operational conditions, the degree of acetylation was 15.26 mol of acetic acid/100 mol of galacturonic acid, in between those determined in the optimum for orange peels and sugar beet pulp (5.56 and 50.66 mol of acetic acid/100 mol of galacturonic acid, respectively) [28,29].

The non-volatile impurities were 18.97 g/100 g NVC, in range with those reported for lemon and orange peels and sugar beet pulp (15–18 g/100 g NVC) [27,28,29]. However, further analysis of the liquors allowed concluding that in MPs, the ONVCs would be justified by protein and ash, with contents of 2.82 g/100 g WIS (corresponding to a solubilization yield of 24.80 g protein in liquor/100 g protein in WISs) and 2.27 g/100 g WIS (solubilization yield of 67.34 g ash in liquor/100 g ash in WISs), respectively. Sugar beet pulp contains a similar amount of protein to water insoluble melon substrate. However, the generally lower protein yields in the recovered liquors (3.95 to 27.09 g protein in liquor/100 g protein in sugar beet pulp at temperatures from 140 to 180 °C) could explain the lower ONVCs reported previously for this raw material [29].

#### 3.2.2. Antioxidant Potential

Another aspect assessed in this work was the antioxidant potential of the liquors from MPs. Phenolic compounds are commonly recovered from fruits and vegetables by aqueous–organic extractions, but an important part of the phenols contained in these raw materials are not extractable by conventional methods. They are called macromolecular antioxidants and include high molecular weight phenols and the low molecular weight ones linked to macromolecules like pectin [58,59]. Hence, there is interest in studying new technologies for the recovery of phenolic compounds in pectin-rich raw materials, since its solubilization could allow access to a macromolecular fraction and thereby increasing its extraction performance.

In this context, this study proposes the hydrothermal treatment, since it has been previously studied for the same purpose with several raw materials such as vine shoots, spent coffee grounds, peanut and hazelnut shells, pomegranate and mango peels and purple corn cob, among others [30,31,49,52,60,61,62]. According to the literature data, the recovery of phenolic compounds by this technology could be achieved by partial lignin depolymerization and by the hydrolysis of low molecular weight phenols bound to oligosaccharides [49,63].

**Figure 4:**
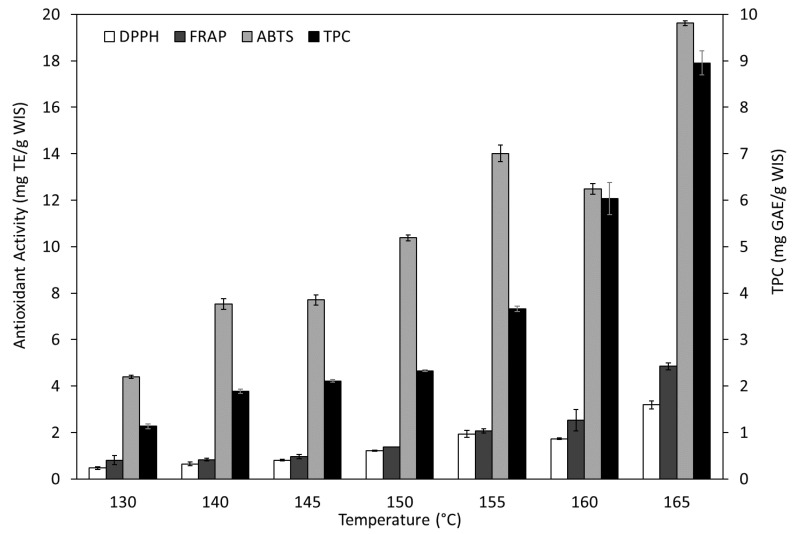
Effects of the autohydrolysis temperature on the total phenolic content (TPC) and antioxidant capacity of the extracted liquors by DPPH (α,α-diphenyl-β-picrylhydrazyl), ABTS (2,2-azino-bis-3-ethylbenzothiazoline-6-sulphonic acid) and FRAP (ferric reducing antioxidant power) assays.

With the aim of knowing the impact of hydrothermal treatment on WISs, the total phenolic content (TPC) and antioxidant activities (DPPH, FRAP and ABTS assays) of all the autohydrolysis extracts obtained has been determined. As can be seen in Figure 4, both TPC and activities increased with temperature, reaching the highest values at the highest temperature studied (TPC of 8.96 mg GAE/g WIS and activities of 3.19, 4.85 and 19.63 mg TE/g WIS determined by DPPH, FRAP and ABTS methods, respectively). Moreover, all the antioxidant activities assayed are in good correlation with the TPC, with R^2^ of 0.89, 0.96 and 0.84 for DPPH, FRAP and ABTS, respectively.

According to Pérez-Jiménez and Saura-Calixto [58], the melon peel of the “Piel de sapo” variety presents a polyphenol content of 9.68 mg GAE/g dw (dry weight) peels, of which 67% are non-extractable. On this basis, the autohydrolysis treatment at 165 °C would have extracted 93% of the total amount contained in the raw material. Although it would be feasible to improve the extraction yield increasing treatment temperatures, with the reported information, it seems reasonable to assume that 165 °C could be close to the optimal treatment temperature for the recovery of phenols. However, due to the typical variability of this kind of raw materials, to confirm this assumption still requires a more exhaustive characterization.

At the optimal temperature selected for the production of pectic oligosaccharides, 140 °C, the phenolic content and antioxidant activities were much lower (TPC: 1.89 mg GAE/g WIS, DPPH: 0.65 mg TE/g WIS, FRAP: 0.83 mg TE/g WIS and ABTS: 7.52 mg TE/g WIS). The TPC was lower than the total extractable polyphenols (3.16 mg GAE/g MP) determined by Pérez-Jiménez and Saura-Calixto [58] with the same melon variety, and within the range provided for the peels of other melon varieties when the extraction was carried out using conventional solvents (1.11–7.03 mg GAE/g MP) [14,40,42]. Certain studies focused on the co-production of oligosaccharides and phenols in aqueous media from peanut shells, spent coffee grounds, chestnut shells, or purple corn cob resulted in higher TPC contents; but in all these cases, given the different nature of the substrates, a considerably higher treatment severity was required to reach the optimal extraction efficiency [52,62,64,65]. The recovery of the phenolic compounds that remain in the solid fraction after melon peel treatment at 140 °C is key for the complete valorization of melon by-products. With this aim, a sequential second stage is currently underway in our laboratories and it will be addressed in future works.

Further HPLC–DAD–MS/MS analysis of the extracts allowed the identification and quantification of three phenolic acids. The main compound detected was 4 hydroxybenzoic acid, with a content of 7.85 µg/g WIS, followed by salicylic and p-coumaric acids (0.51 and 0.12 µg/g WIS, respectively), as shown in Figure S1. This finding is in agreement with previous studies, where hydroxybenzoic acids were also predominant [8,58]. However, other compounds such as gallic acid, catechin or flavones, that were not found in autohydrolysis liquors, were reported in important quantities in melon by-product hydro-alcoholic extracts [8,14,66]. These differences could be associated with the melon variety, the state of ripening, the extraction techniques or analytical methodologies.

### 3.3. Pectin Recovery from Autohydrolysis Liquor

Pectin was recovered and purified from the autohydrolysis liquor obtained at 140 °C by precipitation using an ethanol concentration of 75% *v/v*. As shown in Table 2, the pectin yield was 16.59 g/100 g WIS, in dry weight, with a galacturonic acid content of 55.41% (91.23% of the galacturonic acid present in the original liquor). When comparing with the citric acid extraction, greatly differing melon peel pectin yields (3.24–29.48%) and lower galacturonic acid contents (indicating lower pectin purity) (47–48%) were reported [19,20]. Concerning similar raw materials, several autohydrolysis studies found a wide range in the galacturonic acid content of the obtained pectin, with higher values for the pomelo peels (76.62%) [54] and lower for apple pomace (48%) [55], both of which presented moderately higher pectin yields than this work (19.6 and 17.55%, respectively). Regarding other OSs, GalOS and AraOS also achieved good recoveries, both close to 70%, whereas the other oligosaccharides remained mostly in the liquid phase.

The composition of the precipitated pectin can give more insight into the structure of the solubilized oligosaccharides. A recent study shows that hot water treatments are efficient for the extraction of the HG region of pectin with rhamnose contents of 0.5–0.6%, indicating a low RG-I content [69]. Our results confirm this finding, by increasing HGs and decreasing RG-I in the precipitated pectin, meaning that some of the branched oligosaccharides solubilized would be too degraded to be recovered by precipitation.

Moreover, the sugar ratios can also help to obtain information on the polymeric level [68,70], since sugar ratio 1 measures of the linearity of pectin, sugar ratio 2 indicates the contribution of RG-I to the pectin population and sugar ration 3 compares the amount of RG-I side-chain sugars to rhamnose [68]. In this case, the higher sugar ratio 1 in the precipitate proves the recuperation of the more linear region pectin, with a lower RG-I content (according to the sugar ratio 2 calculated value); whereas the high sugar ratio 3 indicates that the recovered RG region would be highly branched with GalOS and AraOS. Furthermore, the lack of XOS, ManOS and GOS shows that the co-extracted hemicelluloses and cellulosic oligomers were not precipitated with the pectin. A similar linearity and RG-I content, but less branching (sugar ratios of 5.9, 0.01 and 11.38) has also been reported in apple pomace pectin recovered by hydrothermal treatment at the same temperature [55].

Besides oligosaccharides, the recovered pectin contained a substantial amount of protein and some phenolic compounds (6.10 and 0.33 g/100 g dw, respectively), with recovery yields of 35.94 and 29.21%. Moreover, another important parameter to consider would be the OS/MS ratio, which increased from 7.56 to 104.15, with the recovered pectin having a monosaccharide concentration of just 0.67 g/100 g dw.

### 3.4. Structural Characterization

The properties of the recovered oligosaccharides are conditioned not only by their chemical composition but also by their structure, such as the degree of substitution or molecular weight distribution [34,71]. Therefore, a more detailed analysis of the final product can give more insight into its potential applications [71]. Several analytical techniques are often used in the literature to assess the effect of a treatment on the structure of the resulting oligosaccharides. For instance, FTIR and nuclear magnetic resonance (NMR) can be used to confirm the presence of characteristic functional groups or bonds [34,71,72], SEC allows for the determination of molecular weights and polydispersity index [26,32,70,71] and HPAEC–PAD and MALDI-TOF MS are commonly used to determine degrees of polymerization in oligosaccharides, with MALDI-TOF MS also being able to identify the composition of oligomers based on their molecular weight [26]. In this context, the liquors obtained in this study at 140 °C were freeze-dried and analyzed by complementary techniques: MALDI-TOF MS and HPAEC–PAD; and both the freeze-dried liquors and the pectin recovered by precipitation were analyzed by FTIR.

**Figure 5:**
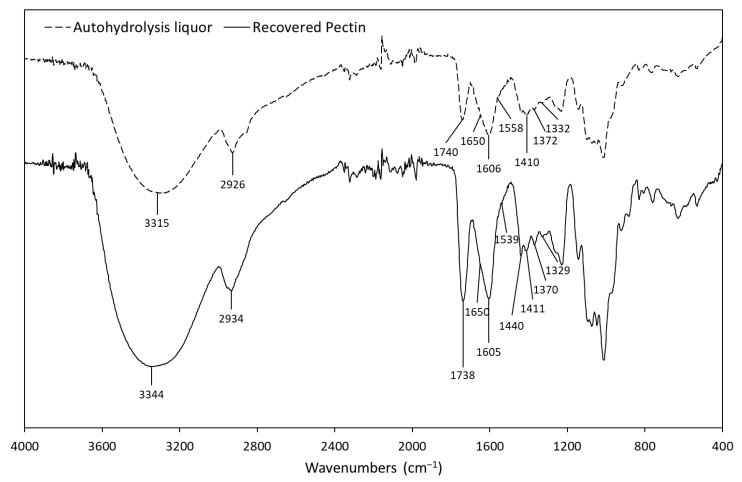
FTIR spectra recorded for the autohydrolysis liquors and recovered pectin.

Both FTIR spectra (Figure 5) show similar bands to those of pectins found in the bibliography, especially the purified pectin with more intense peaks [19,20,31,70,72,73]. The peak at 3200–3500 cm^−1^ corresponds to OH stretching as a result of hydrogen bonding in pectin [19,31,71]. The peak between 2800 and 3000 cm^−1^ is related to stretching and bending vibrations of alkyl groups in the carbohydrates making up the pectin [20,31,71]. The observation of methyl esterified carboxyl groups (-COOCH_3_, at 1738–1740 cm^−1^) and free carboxyl groups (-COO^−^, at 1605–1606 cm^−1^) confirmed the presence of pectin with some degree of methyl esterification [19,31,72]. Using these two peaks and the calibration curve determined by Manrique and Lajolo [74], the degree of methyl esterification (DM) of the OGalA extracted in this work was estimated to be 41% (molar) for the autohydrolysis liquor and 50% for the recovered pectin. However, these values might actually be higher, since the considerable protein content of these extracts could be showing peaks at 1650 and 1539–1558 cm^−1^, overlapping with the 1606 cm^−1^ band [70]. The increase in DM when recovering the pectin is in agreement with the literature regarding hot water treatments, which tend to extract pectins with high DM [69]. Free carboxyl groups also show weaker bands at 1410–1440 cm^−1^ related to symmetric stretching [19,73,75]. Other weaker and less relevant bands are the ones at 1370–1372 cm^−1^, that could be caused by CH stretching vibrations [72,75] and the one at 1329–1332 cm^−1^, that could be related to the COO- functional group in pectin [20]. The patterns between 1200 and 800 cm^−1^ represent the “finger print” area, which is unique to a compound, making its interpretation a complicated matter [19,73].

**Figure 6:**
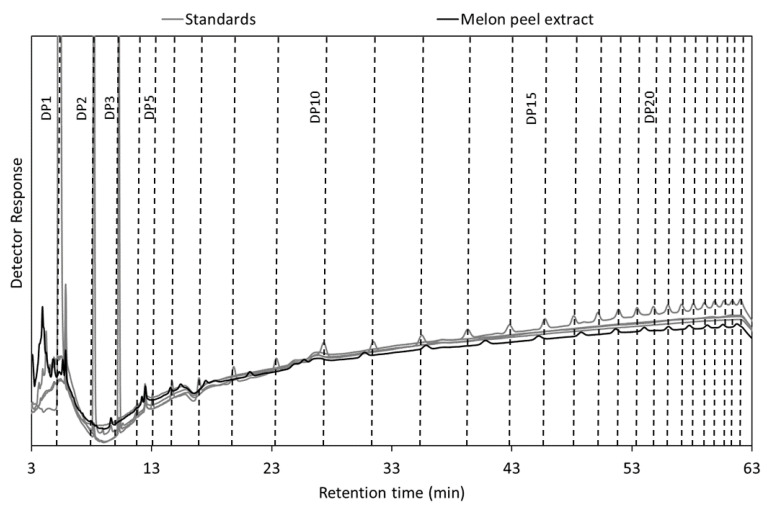
HPAEC–PAD of melon peel extracts and standards (DP1, DP2, DP3 and PGA).

The degree of polymerization (DP) of the oligogalacturonides was determined by MALDI-TOF MS. Table 3 displays the mass signals in MALDI-TOF spectra and suggested oligomeric structures (all compounds were identified as sodium or potassium adducts). The MALDI-TOF MS data confirmed the presence of a wide variety of complex oligomers, with the main products being combinations of galaturonic acid in the range of 1–11 with neutral sugars (mainly hexoses, pentoses and rhamnose) and substituted by acetyl and methyl groups. Overall, the complex chemical structure determined for the oligomeric compounds obtained from the melon peels is similar to what it has been reported for other oligosaccharides obtained by enzymatic hydrolysis from lemon peels [76]. Furthermore, HPAEC–PAD analysis (Figure 6) showed OGalA with DP from 5 to over 20 (using commercial polygalacturonic acid (PGA) as standard).

### 3.5. Valorization of the Autohydrolysis Spent Solids

Melon peels can be considered an abundant, renewable and cheap source of biomass whose complete valorization to obtain various biobased products is an interesting strategy from an economic and environmental point of view. In this context, the potential application of the spent solid recovered from the selected hydrothermal treatment has been evaluated. It should be noted that operating at 140 °C, the solid yield was 72.88%, hence the importance of its valorization. The main components of the autohydrolyzed solid were glucan and Klason lignin, with values of 31.26 and 30.59 g/100 g spent solid, respectively, and percentages of recovery for both fractions of 93% and close to 100%. These results are better than those previously reported for the autohydrolysis of other raw materials rich in pectin. When sugar beet pulp and orange and lemon peels were treated under similar severities, the percentages of glucan recoveries were in the range of 75–80% and in the case of citric peels, lignin recoveries close to 92% were achieved [27,28,29]. The selected operational temperature allowed reaching high pectic oligosaccharide extraction, whereas, as could be expected, other hemicellulosic fractions remained in the spent solid; since according to the literature their solubilization requires higher temperatures [34,52,77]. This leads to the fact that small amounts of xylan, galacturonan and galactan were also detected in the autohydrolyzed solid (6.90, 5.79 and 2.78%, respectively), which are followed by acetyl groups, mannan and arabinosyl substituents, with values lower than 2%. The behavior pattern of these compounds during autohydrolysis could be related with structural features, since they could be part of some hemicellulosic polysaccharides or be linked to the galacturonic acid of pectin.

Taking into account the chemical composition of the spent solid recovered in the autohydrolysis of melon peels at 140 °C, it would be plausible to implement a second autohydrolysis step to obtain a solid fraction enriched in cellulose with potential applications, and a liquid fraction with a higher phenolic content [78]. For instance, this cellulose would be suitable for the biotechnological production of fermentable sugars, or for the production of nanocellulose [13]. In this work, the digestibility of the spent solid was assessed without further processing, by subjecting it to an enzymatic hydrolysis using an enzyme cocktail, specifically a mixture of endopolygalacturonase, cellulase and β-glucosidase. Under the experimental conditions selected (see in the Materials and Methods Section), after 24 h of saccharification, 79.83% of the glucan was solubilized, yielding a solution with 22.41 g/L of glucose. Other compounds, such as galactose and galacturonic acid, were also detected in the reaction medium, but in much lower amounts (3.29 and 2.7 g/L, respectively). Further increases in the hydrolysis time led to glucan conversions into glucose up to 87.65% after 48 h.

## 4. Conclusions

Pectic oligosaccharides with antioxidant activity were recovered from melon by-products using an environmentally friendly process. The raw material was subjected to a water extraction to release free sugars and the remaining solid was treated by autohydrolysis in non-isothermal conditions. The selected temperature for this treatment was 140 °C (severity of 2.03), achieving a liquor with a total oligosaccharide content of 15.24 g/100 g WIS, of which 10.07 g/100 g WIS were OGalA. A structural characterization confirmed the presence of OGalA with a degree of methyl esterification of at least 41% and a wide range of polymerization degrees. These liquors also exhibited antioxidant activity and contained a considerable amount of protein (2.82 g/100 g WIS), with a negligible amount of non-volatile impurities. The pectic oligosaccharides were successfully recovered by precipitation, producing a mostly linear pectin with 55.41% of galacturonic acid. In conclusion, an environmentally friendly process was developed for the valorization of melon by-products, through the production of pectic oligosaccharides with protein content and antioxidant activity, and potential applications in the food and pharmaceutical industries.

## Figures and Tables

**Table 1:** Chemical composition of the aqueous extracts and the water-insoluble solids (WISs) on a dry basis.

Component	Content
**Extracts** (g/100 g extracts)	
Glucose	36.40 ± 3.18
Sucrose	5.35 ± 0.24
Fructose	45.79 ± 0.26
Uronic Acids	2.79 ± 0.04
Citric Acid	5.59 ± 1.00
TPC	0.89 ± 0.02
Protein	10.81 ± 1.05
**WIS** (g/100 g WIS)	
Glucan	24.54 ± 0.10
Xylan	4.89 ± 0.21
Galactan	3.31 ± 0.21
Mannan	1.54 ± 0.04
Arabinosyl S.	1.56 ± 0.11
Acetyl Groups	2.05 ± 0.35
Galacturonan	11.99 ± 0.69
Klason lignin	19.96 ± 1.72
Protein	11.36 ± 1.86
Ash	3.36 ± 0.03
mg/100 g WIS	
Iron	8.74 ± 3.97
Potassium	863.01 ± 109.81
Manganese	1.83 ± 0.36
Magnesium	218.73 ± 50.57

**Table 2:** Chemical characterization of the selected autohydrolysis liquor and the precipitated pectin.

	Autohydrolysis Liquor	Recovered Pectin	Recovery Yield
	Chemical Composition (g/100 g dw)		
NVC (g/100 g WIS)	23.22	16.59	71.43
GOS	2.50	0.14	4.07
XOS	1.40	0.00	0.00
GalOS	8.15	8.05	70.50
ManOS	1.97	0.00	0.00
AraOS	4.62	4.37	67.47
RhaOS	1.56	0.51	23.37
Ac-OS	2.05	1.57	54.68
OGalA	43.38	55.41	91.23
Protein	12.13	6.10	35.94
Ash	9.75	6.88	50.40
TPC	0.81	0.33	29.21
**Other Parameters**			
Moisture (%)	98.65	7.02	
OS/MS ratio (*w*/*w*)	7.56	104.15	
DA (molar %)	15.26	9.15	
HG * (molar %)	62.34	77.75	
RG-I * (molar %)	27.71	22.03	
HG/RG-I	2.25	3.53	
Sugar Ratios ** (*w*/*w*)			
1 $\frac{\mathrm{GalA}}{\mathrm{Rha}+\mathrm{Ara}+\mathrm{Gal}+\mathrm{Xyl}}$	2.76	4.29	
2 $\frac{\mathrm{Rha}}{\mathrm{GalA}}$	0.04	0.01	
3 $\frac{\mathrm{Ara}+\mathrm{Gal}}{\mathrm{Rha}}$	8.17	24.27	

* Determined as defined by M’sakni et al. [67]. ** Calculated according to Houben et al. [68]. GalA: galacturonic acid; Rha: rhamnose; Gal: galactose; Ara: arabinose; Xyl: xylose; HG: homogalacturonan; RG: rhamnogalacturonan.

**Table 3:** MALDI-TOF results and suggested structures of autohydrolysis liquors at 140 °C.

*m/z*	Structure
648.52	Pent_2_GalAAc_4_Na
847.58	HexPentRhaGalA_2_MeNa
980.57	Pent_3_GalA_3_MeNa
1174.34	HexGalA_4_Me_4_Ac_5_Na
1463.65	Hex_2_GalA_6_Me_3_Na
1603.34	GalA_8_Me_5_Ac_2_Na
1790.94	Pent_2_GalA_7_Me_3_Ac_5_Na
1889.26	Pent_3_GalA_8_Me_3_Na
2409.23	Hex_1_Pent_3_GalA_9_Me_4_Ac_4_Na
2641.94	Hex_4_PentGalA_10_AcK
3498.19	Hex_4_Pent_2_Rha_3_GalA_11_Me_3_Ac_3_Na

Hex: hexose; Pent: pentose; Rha: rhamnose; Ac: acetyl group; GalA: galacturonic acid; Me: methyl group.

## Supplementary Materials

The following are available online at https://www.mdpi.com/2304-8158/9/11/1702/s1, Figure S1: HPLC UV/VIS chromatograms of melon peel autohydrolysis liquors at 140 °C.

## References

[1] FAO (2019). The State of Food and Agriculture 2019. Moving Forward on Food Loss and Waste Reduction.

[2] Barilla Center for Food & Nutrition (2012). Food Waste: Causes, Impacts and Proposals.

[3] Stenmarck Å., Jensen C., Quested T., Moates G. (2016). Estimates of European Food Waste Levels.

[4] Fava F., Totaro G., Diels L., Reis M., Duarte J., Poggi-varaldo M., Ferreira B.S., Carioca O.B. (2015). Biowaste biorefinery in Europe: Opportunities and research & development needs. New Biotechnol..

[5] Banerjee J., Singh R., Vijayaraghavan R., Macfarlane D., Patti A.F., Arora A. (2017). Bioactives from fruit processing wastes: Green approaches to valuable chemicals. Food Chem..

[6] European Commission (2018). Directive (EU) 2018/850 Amending Directive 1999/31/EC on the Landfill of Waste.

[7] European Commission (2018). Directive (EU) 2018/851 Amending Directive 2008/98/EC on Waste.

[8] Mallek-Ayadi S., Bahloul N., Kechaou N. (2017). Characterization, phenolic compounds and functional properties of *Cucumis melo* L. peels. Food Chem..

[9] FAO Faostat Database. www.fao.org/faostat.

[10] Aguayo E., Escalona V.H., Artés F.A. (2004). Metabolic Behavior and Quality Changes of Whole and Fresh Processed Melon. J. Food Sci..

[11] Fundo J.F., Miller F.A., Garcia E., Santos J.R., Silva C.L.M., Brandão T.R.S. (2018). Physicochemical characteristics, bioactive compounds and antioxidant activity in juice, pulp, peel and seeds of Cantaloupe melon. J. Food Meas. Charact..

[12] Gómez-García R., Campos D.A., Aguilar C.N., Madureira A.R., Pintado M. (2020). Valorization of melon fruit (*Cucumis melo* L.) by-products: Phytochemical and Biofunctional properties with Emphasis on Recent Trends and Advances. Trends Food Sci. Technol..

[13] Rico X., Gullón B., Alonso J.L., Yáñez R. (2020). Recovery of high value-added compounds from pineapple, melon, watermelon and pumpkin processing by-products: An overview. Food Res. Int..

[14] Rolim P.M., Fidelis G.P., Padilha C.E.A., Santos E.S., Rocha H.A.O., Macedo G.R. (2018). Phenolic profile and antioxidant activity from peels and seeds of melon (*Cucumis melo* L. var. *reticulatus*) and their antiproliferative effect in cancer cells. Braz. J. Med. Biol. Res..

[15] Rolim P.M., de Oliveira Júnior S.D., Mendes de Oliveira A.C.S., dos Santos E.S., de Macedo G.R. (2018). Nutritional value, cellulase activity and prebiotic effect of melon residues (*Cucumis melo* L. *reticulatus* group) as a fermentative substrate. J. Food Nutr. Res..

[16] Toledo N.M.V., Mondoni J., Harada-Padermo S.S., Vela-Paredes R.S., Berni P.R.A., Selani M.M., Canniatti Brazaca S.G. (2019). Characterization of apple, pineapple, and melon by-products and their application in cookie formulations as an alternative to enhance the antioxidant capacity. J. Food Process. Preserv..

[17] Wang F., Li H., Zhao H., Zhang Y., Qiu P., Li J., Wang S. (2018). Antidiabetic Activity and Chemical Composition of Sanbai Melon Seed Oil. Evidence-Based Complement. Altern. Med..

[18] Silva M.A., Albuquerque T.G., Alves R.C., Oliveira M.B.P.P., Costa H.S. (2020). Melon (*Cucumis melo* L.) by-products: Potential food ingredients for novel functional foods?. Trends Food Sci. Technol..

[19] Raji Z., Khodaiyan F., Rezaei K., Kiani H., Hosseini S.S. (2017). Extraction optimization and physicochemical properties of pectin from melon peel. Int. J. Biol. Macromol..

[20] Muthukumaran C., Banupriya L., Harinee S., Sivaranjani S., Sharmila G., Rajasekar V., Kumar N.M. (2017). Pectin from muskmelon (*Cucumis melo* var. *reticulatus*) peels: Extraction optimization and physicochemical properties. 3 Biotech..

[21] Naqash F., Masoodi F.A., Rather S.A., Wani S.M., Gani A. (2017). Emerging concepts in the nutraceutical and functional properties of pectin—A Review. Carbohydr. Polym..

[22] Adetunji L.R., Adekunle A., Orsat V., Raghavan V. (2017). Advances in the pectin production process using novel extraction techniques: A review. Food Hydrocoll..

[23] Gullón B., Gómez B., Martínez-Sabajanes M., Yáñez R., Parajó J.C., Alonso J.L. (2013). Pectic oligosaccharides: Manufacture and functional properties. Trends Food Sci. Technol..

[24] Wang W., Chen W., Zou M., Lv R., Wang D., Hou F., Feng H., Ma X., Zhong J., Ding T. (2018). Applications of power ultrasound in oriented modification and degradation of pectin: A review. J. Food Eng..

[25] Kumar M., Tomar M., Saurabh V., Mahajan T., Punia S., Contreras M., del Mar Contreras M., Rudra S.G., Kaur C., Kennedy J.F. (2020). Emerging trends in pectin extraction and its anti-microbial functionalization using natural bioactives for application in food packaging. Trends Food Sci. Technol..

[26] Babbar N., Dejonghe W., Gatti M., Sforza S., Elst K. (2015). Pectic oligosaccharides from agricultural by-products: Production, characterization and health benefits. Crit. Rev. Biotechnol..

[27] Gómez B., Gullón B., Yáñez R., Parajó J.C., Alonso J.L. (2013). Pectic Oligosacharides from Lemon Peel Wastes: Production, Purification, and Chemical Characterization. J. Agric. Food Chem..

[28] Martínez M., Yáñez R., Alonso J.L., Parajó J.C. (2010). Chemical Production of Pectic Oligosaccharides from Orange Peel Wastes. Ind. Eng. Chem. Res..

[29] Martínez M., Gullón B., Schols H.A., Alonso J.L., Parajó J.C. (2009). Assessment of the Production of Oligomeric Compounds from Sugar Beet Pulp. Ind. Eng. Chem. Res..

[30] Talekar S., Patti A.F., Vijayaraghavan R., Arora A. (2018). An integrated green biorefinery approach towards simultaneous recovery of pectin and polyphenols coupled with bioethanol production from waste pomegranate peels. Bioresour. Technol..

[31] Banerjee J., Singh R., Vijayaraghavan R., MacFarlane D., Patti A.F., Arora A. (2018). A hydrocolloid based biorefinery approach to the valorisation of mango peel waste. Food Hydrocoll..

[32] Dávila I., Gordobil O., Labidi J., Gullón P. (2016). Assessment of suitability of vine shoots for hemicellulosic oligosaccharides production through aqueous processing. Bioresour. Technol..

[33] Garrote G., Domínguez H., Parajó J.C. (1999). Mild autohydrolysis: An environmentally friendly technology for xylooligosaccharide production from wood. J. Chem. Technol. Biotechnol..

[34] Dávila I., Gullón B., Alonso J.L., Labidi J., Gullón P. (2019). Vine shoots as new source for the manufacture of prebiotic oligosaccharides. Carbohydr. Polym..

[35] López M., Penín L., Vila C., Santos V., Parajó J.C. (2019). Multi-Stage Hydrothermal Processing of Eucalyptus Globulus Wood: An Experimental Assessment. J. Wood Chem. Technol..

[36] Grajek W., Olejnik A., Sip A. (2005). Probiotics, prebiotics and antioxidants as functional foods. Acta Biochim. Pol..

[37] Willats W.G.T., Knox J.P., Mikkelsen J.D. (2006). Pectin: New insights into an old polymer are starting to gel. Trends Food Sci. Technol..

[38] De Paulo Farias D., de Araújo F.F., Neri-Numa I.A., Pastore G.M. (2019). Prebiotics: Trends in food, health and technological applications. Trends Food Sci. Technol..

[39] Sagar N.A., Pareek S., Sharma S., Yahia E.M., Lobo M.G. (2018). Fruit and Vegetable Waste: Bioactive Compounds, Their Extraction, and Possible Utilization. Compr. Rev. Food Sci. Food Saf..

[40] Morais D.R., Rotta E.M., Sargi S.C., Schmidt E.M., Bonafe E.G., Eberlin M.N., Sawaya A.C.H.F., Visentainer J.V. (2015). Antioxidant activity, phenolics and UPLC-ESI(-)-MS of extracts from different tropical fruits parts and processed peels. Food Res. Int..

[41] Guo C., Yang J., Wei J., Li Y., Xu J., Jiang Y. (2003). Antioxidant activities of peel, pulp and seed fractions of common fruits as determined by FRAP assay. Nutr. Res..

[42] Ismail H.I., Chan K.W., Mariod A.A., Ismail M. (2010). Phenolic content and antioxidant activity of cantaloupe (cucumis melo) methanolic extracts. Food Chem..

[43] Miller F.A., Fundo J.F., Garcia E., Santos J.R., Silva C.L.M., Brandão T.R.S. (2020). Physicochemical and Bioactive Caracterisation of Edible and Waste Parts of “Piel de Sapo” Melon. Horticulturae.

[44] Míguez B., Gómez B., Gullón P., Gullón B., Alonso J.L. (2016). Pectic Oligosaccharides and Other Emerging Prebiotics. Probiotics and Prebiotics in Human Nutrition and Health.

[45] Lavoie J.M., Capek-Menard E., Gauvin H., Chornet E. (2010). Quality pulp from mixed softwoods as an added value coproduct of a biorefinery. Ind. Eng. Chem. Res..

[46] Blumenkrantz N., Asboe-Hansen G. (1973). New method for quantitative determination of uronic acids. Anal. Biochem..

[47] Martínez M., Gullón B., Yáñez R., Alonso J.L., Parajó J.C. (2009). Direct Enzymatic Production of Oligosaccharide Mixtures from Sugar Beet Pulp: Experimental Evaluation and Mathematical Modeling. J. Agric. Food Chem..

[48] Singleton V.L., Rossi J.A. (1965). Colorimetry of Total Phenolics with Phosphomolybdic-Phosphotungstic Acid Reagents. Am. J. Enol. Vitic..

[49] Gullón B., Eibes G., Moreira M.T., Dávila I., Labidi J., Gullón P. (2017). Antioxidant and antimicrobial activities of extracts obtained from the refining of autohydrolysis liquors of vine shoots. Ind. Crops Prod..

[50] Gómez B., Gullón B., Remoroza C., Schols H.A., Parajó J.C., Alonso J.L. (2014). Purification, characterization, and prebiotic properties of pectic oligosaccharides from orange peel wastes. J. Agric. Food Chem..

[51] Morais D.R., Rotta E.M., Sargi S.C., Bonafe E.G., Suzuki R.M., Souza N.E., Matsushita M., Visentainer J.V. (2017). Proximate composition, mineral contents and fatty acid composition of the different parts and dried peels of tropical fruits cultivated in Brazil. J. Braz. Chem. Soc..

[52] Rico X., Gullón B., Alonso J.L., Parajó J.C., Yáñez R. (2018). Valorization of peanut shells: Manufacture of bioactive oligosaccharides. Carbohydr. Polym..

[53] Klinchongkon K., Khuwijitjaru P., Wiboonsirikul J., Adachi S. (2015). Extraction of Oligosaccharides from Passion Fruit Peel by Subcritical Water Treatment. J. Food Process Eng..

[54] Liew S.Q., Teoh W.H., Tan C.K., Yusoff R., Ngoh G.C. (2018). Subcritical water extraction of low methoxyl pectin from pomelo *(Citrus grandis* (L.) Osbeck) peels. Int. J. Biol. Macromol..

[55] Wang X., Lü X. (2014). Characterization of pectic polysaccharides extracted from apple pomace by hot-compressed water. Carbohydr. Polym..

[56] Muñoz-Almagro N., Valadez-Carmona L., Mendiola J.A., Ibáñez E., Villamiel M. (2019). Structural characterisation of pectin obtained from cacao pod husk. Comparison of conventional and subcritical water extraction. Carbohydr. Polym..

[57] Li W.J., Fan Z.G., Wu Y.Y., Jiang Z.G., Shi R.C. (2019). Eco-friendly extraction and physicochemical properties of pectin from jackfruit peel waste with subcritical water. J. Sci. Food Agric..

[58] Pérez-Jiménez J., Saura-Calixto F. (2018). Fruit peels as sources of non-extractable polyphenols or macromolecular antioxidants: Analysis and nutritional implications. Food Res. Int..

[59] Pérez-Jiménez J., Saura-Calixto F. (2015). Macromolecular antioxidants or non-extractable polyphenols in fruit and vegetables: Intake in four European countries. Food Res. Int..

[60] Ballesteros L.F., Ramirez M.J., Orrego C.E., Teixeira J.A., Mussatto S.I. (2017). Optimization of autohydrolysis conditions to extract antioxidant phenolic compounds from spent coffee grounds. J. Food Eng..

[61] Pérez-Armada L., Rivas S., González B., Moure A. (2019). Extraction of phenolic compounds from hazelnut shells by green processes. J. Food Eng..

[62] Gullón P., Eibes G., Lorenzo J.M., Pérez-Rodríguez N., Lú-Chau T.A., Gullón B. (2020). Green sustainable process to revalorize purple corn cobs within a biorefinery frame: Co-production of bioactive extracts. Sci. Total Environ..

[63] Leschinsky M., Zuckerstätter G., Weber H.K., Patt R., Sixta H. (2008). Effect of autohydrolysis of Eucalyptus globulus wood on lignin structure. Part 1: Comparison of different lignin fractions formed during water prehydrolysis. Holzforschung.

[64] Ballesteros L.F., Teixeira J.A., Mussatto S.I. (2017). Extraction of polysaccharides by autohydrolysis of spent coffee grounds and evaluation of their antioxidant activity. Carbohydr. Polym..

[65] Gullón B., Eibes G., Dávila I., Moreira M.T., Labidi J., Gullón P. (2018). Hydrothermal treatment of chestnut shells (*Castanea sativa*) to produce oligosaccharides and antioxidant compounds. Carbohydr. Polym..

[66] Vella F.M., Cautela D., Laratta B. (2019). Characterization of Polyphenolic Compounds in Cantaloupe Melon By-Products. Foods.

[67] M’sakni N.H., Majdoub H., Roudesli S., Picton L., Le Cerf D., Rihouey C., Morvan C. (2006). Composition, structure and solution properties of polysaccharides extracted from leaves of Mesembryanthenum crystallinum. Eur. Polym. J..

[68] Houben K., Jolie R.P., Fraeye I., Van Loey A.M., Hendrickx M.E. (2011). Comparative study of the cell wall composition of broccoli, carrot, and tomato: Structural characterization of the extractable pectins and hemicelluloses. Carbohydr. Res..

[69] Mao G., Wu D., Wei C., Tao W., Ye X., Linhardt R.J., Orfila C., Chen S. (2019). Reconsidering conventional and innovative methods for pectin extraction from fruit and vegetable waste: Targeting rhamnogalacturonan I. Trends Food Sci. Technol..

[70] Kpodo F.M., Agbenorhevi J.K., Alba K., Bingham R.J., Oduro I.N., Morris G.A., Kontogiorgos V. (2017). Pectin isolation and characterization from six okra genotypes. Food Hydrocoll..

[71] Grassino A.N., Barba F.J., Brnčić M., Lorenzo J.M., Lucini L., Brnčić S.R. (2018). Analytical tools used for the identification and quantification of pectin extracted from plant food matrices, wastes and by-products: A review. Food Chem..

[72] Kazemi M., Khodaiyan F., Labbafi M., Saeid Hosseini S., Hojjati M. (2019). Pistachio green hull pectin: Optimization of microwave-assisted extraction and evaluation of its physicochemical, structural and functional properties. Food Chem..

[73] Hosseini S.S., Khodaiyan F., Yarmand M.S. (2016). Optimization of microwave assisted extraction of pectin from sour orange peel and its physicochemical properties. Carbohydr. Polym..

[74] Manrique G.D., Lajolo F.M. (2002). FT-IR spectroscopy as a tool for measuring degree of methyl esterification in pectins isolated from ripening papaya fruit. Postharvest Biol. Technol..

[75] Gnanasambandam R., Proctor A. (2000). Determination of pectin degree of esterification by diffuse reflectance Fourier transform infrared spectroscopy. Food Chem..

[76] Gómez B., Yáñez R., Parajó J.C., Alonso J.L. (2016). Production of pectin-derived oligosaccharides from lemon peels by extraction, enzymatic hydrolysis and membrane filtration. J. Chem. Technol. Biotechnol..

[77] Penín L., Santos V., del Río J.C., Parajó J.C. (2019). Assesment on the chemical fractionation of Eucalyptus nitens wood: Characterization of the products derived from the structural components. Bioresour. Technol..

[78] Bassani A., Fiorentini C., Vadivel V., Moncalvo A., Spigno G. (2020). Implementation of auto-hydrolysis process for the recovery of antioxidants and cellulose from wheat straw. Appl. Sci..

