# Supplementary figures and images for: Environmentally Friendly Hydrothermal Processing of Melon by-Products for the Recovery of Bioactive Pectic-Oligosaccharides
Source: Foods. 2020 Nov 20;9(11):1702. doi: 10.3390/foods9111702 (PMC7699732; doi:10.3390/foods9111702)

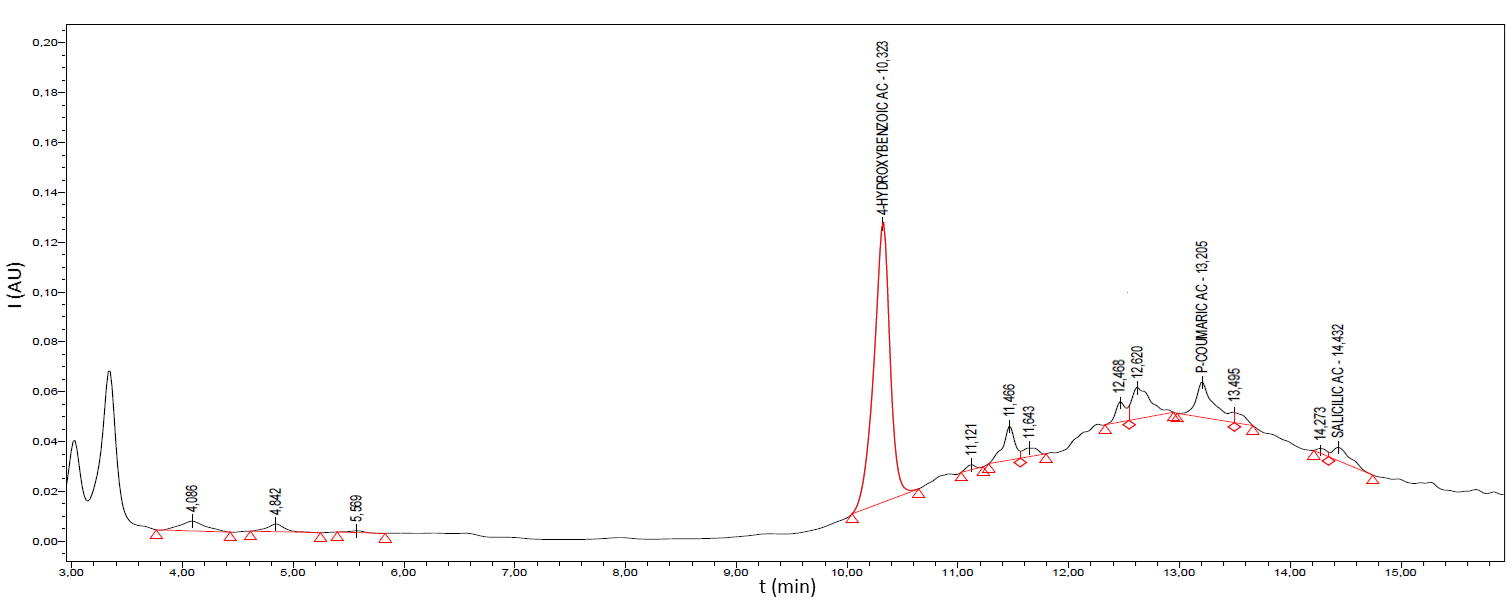

Supplement: Supplementary file 1 [file foods-09-01702-s001.zip › foods-1002692-supplementary.tif]
